# Robust speckle-free imaging using random lasers enhanced by TiN nanoparticles in complex scattering environments

**DOI:** 10.1515/nanoph-2023-0484

**Published:** 2023-11-02

**Authors:** Yuan Wan, Zhihao Li, Zexu Liu, Yang Yang, Hongzhen Wang, Xianlong Liu, Yangjian Cai

**Affiliations:** Shandong Provincial Engineering and Technical Center of Light Manipulations, Shandong Provincial Key Laboratory of Optics and Photonic Device, School of Physics and Electronics, Shandong Normal University, Jinan 250014, China; Joint Research Center of Light Manipulation Science and Photonic Integrated Chip of East China Normal University and Shandong Normal University, East China Normal University, Shanghai 200241, China

**Keywords:** random laser, TiN nanoparticles, speckle-free imaging, low coherence, scattering environments

## Abstract

A light source with narrowband, sufficient brightness, and low spatial coherence is required for certain applications such as optical imaging and free-space optical communication. In this study, our focus was to investigate a novel imaging laser source, specifically a low-threshold random laser enhanced by TiN nanoparticles. The results demonstrate that the random laser spectrum exhibits an impressive bandwidth of 0.23 nm, accompanied by an incredibly low spatial coherence factor of merely 0.15. Due to the low spatial coherence of random laser, the speck contrast is less than 0.02 when the light passes through a scattering system. Notably, when compared to traditional lasers, the use of a random laser yields significantly superior imaging quality in both scatterless and complex scattering environments. This finding highlights the immense potential of the random laser as a narrowband and low spatial coherence laser source for robust speckle-free imaging applications, particularly in environments with intricate scattering phenomena. Furthermore, this breakthrough can be extended to various other domains, including free-space optical communication.

## 1 Introduction

The partially coherent beam has significant potential to reduce the scattering effect of the optical field and the error rate of free-space optical communication. This characteristic renders it highly valuable in various domains such as laser fusion [1], optical imaging [2], quantum optics [3], and free-space optical communication [4]. Currently, a common approach to reducing laser coherence involves the incorporation of optical elements (e.g., rotating ground glass [5–7], liquid crystal light modulator [8, 9]) outside the laser’s resonant cavity. However, this method results in substantial energy loss and necessitates the addition of extra devices. Therefore, there is a need for a partially coherent laser generation method that can achieve coherence modulation within the cavity, offering both high energy and low coherence. Random lasers, which lack a predefined resonant cavity, derive their optical feedback from multiple light scattering within a disordered medium [10–15]. Due to their unique formation mechanism, random lasers exhibit narrowband emission with low coherence, which can be adjusted by altering the size or concentration of scattering particles and the area or shape of the pump light [16]. Consequently, random lasers hold clear advantages in generating partially coherent laser beams.

To achieve high-definition and speckle-free imaging, it is necessary for the light source to possess both high intensity and low coherence. Light-emitting diodes (LEDs) are excellent sources of spatially incoherent light. However, their spectral linewidth is often too wide, and their intensity may be insufficient for certain applications. On the other hand, conventional lasers can produce high intensity and narrow linewidth spectra. Nevertheless, the high coherence of conventional lasers leads to the formation of speckle patterns that can degrade imaging quality. Amplified spontaneous emission (ASE) is a kind of partially coherent light source, which can suppress speckle formation compared with the conventional laser source. However, the spectral linewidth of ASE is much wider than lasers, and the ASE source has a higher spatial coherence than random lasers. This makes that the speckle is clearly visible in the image [17]. Fortunately, random lasers exhibit both narrowband emission, high intensity, and low spatial coherence, making them promising candidates for speckle-free imaging applications [17–20]. In 2012, Cao et al. [17] demonstrated that random lasers serve as high-quality light sources for speckle-free imaging. Additionally, in 2019, Song et al. [18] investigated the use of surface-emitting perovskite random lasers for speckle-free imaging applications. However, dielectric particle-based random lasers commonly suffer from unsatisfactory output light intensity and linewidth. Therefore, further systematic study and improvement are required to enhance the imaging clarity and resolution provided by random lasers in complex environments.

Incorporating plasmonic nanoparticles (NPs) into random gain systems provides an effective means to modulate the radiation characteristics of random lasers, including reducing the laser threshold, enhancing the output intensity, and modulating the emission wavelength, among other effects [21–26]. Currently, gold and silver NPs are the primary plasmonic materials investigated in the field of plasmonic random lasers. However, the intrinsic limitations of gold and silver NPs pose challenges to the development and application of plasmonic random lasers [27]. To overcome these limitations, titanium nitride (TiN) has emerged as a promising alternative due to its favorable plasmonic properties, attributed to its high carrier concentration (approximately 10^22^ cm^−3^), making it well suited for plasmon effects in the visible and near-infrared wavelengths [28–31].

In this article, we conducted an experiment utilizing random lasers enhanced by TiN NPs as the laser source for speckle-free imaging. Our study aimed to systematically investigate the random laser’s ability to withstand interference from the external environment. The results demonstrate that the low spatial coherence and narrowband emission of the random laser contribute to robust speckle-free imaging in complex scattering environments when used as a laser source for imaging.

## 2 Methods

### 2.1 Sample preparation

The random laser used in this article is composed by the DCM dye, TiN NPs, and nematic liquid crystals (NLCs). Details of these materials can be seen in Supporting Information. The DCM provides gain, the NLCs provide strong scattering, and the TiN NPs provide the scattering and localized surface plasmon resonance (LSPR) for the random laser formation. The sample preparation process is as follows: first, 0.5 wt% DCM is dissolved in the NLC solution. Then, TiN NPs are dispersed in this solution to form the DCM-doped nematic liquid crystals with the 1.468 × 10^12^ ml^−1^ of TiN NPs (NPDDNLC) solution. For uniform dispersion of TiN NPs in the mixture, the ultrasonic dispersion process is applied about 20 min at room temperature. Finally, the uniform mixture solution fills into capillary tubes with the inner diameter of 500 µm via the capillary effect to form the NPDDNLC random laser sample.

### 2.2 Optical measurement

**Figure 1:**
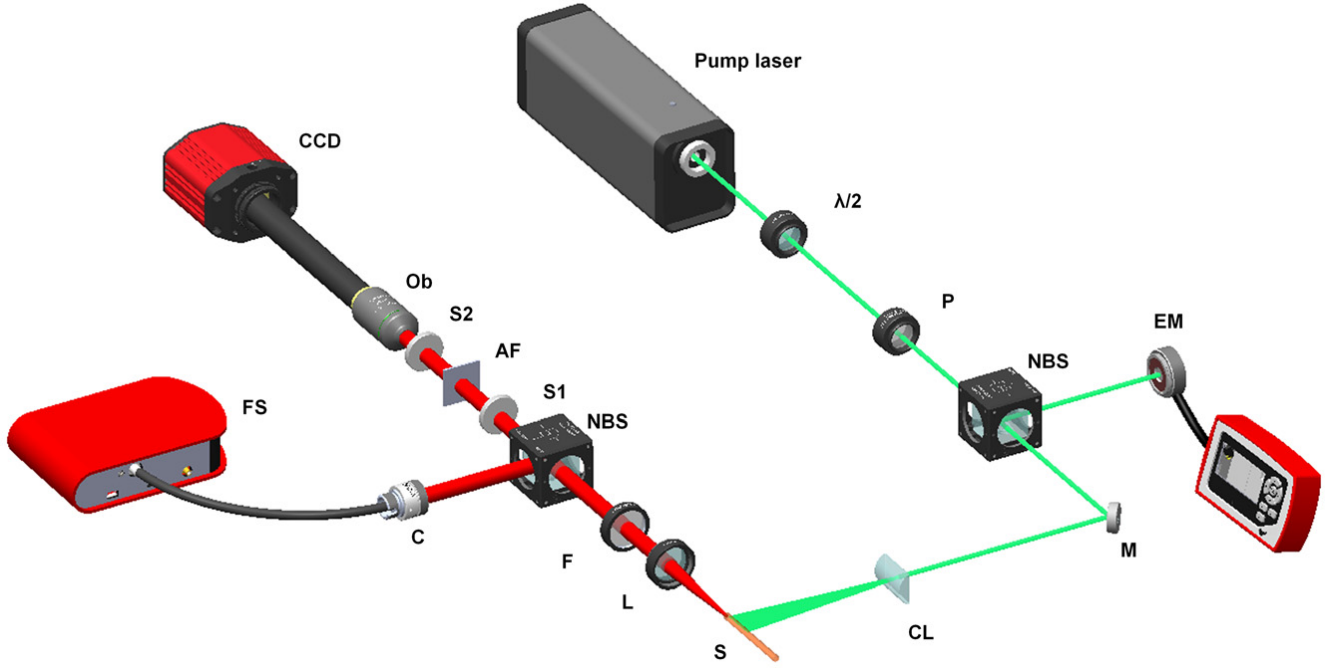
Schematic diagram of the experimental setup.

The experimental setup is shown in Figure 1. The pump source is a frequency doubled Q-switched Nd:YAG pulsed laser (*λ* = 532 nm, BLAZER-2P, Grace Laser Inc.), with a 10 Hz repetition rate and 20 ps pulse duration. The energy and polarization of the pump beam can be varied by a half wave plate (*λ*/2) and a polarizer (*P*). The pump beam is split to two sub-beams with same energy by using a beam splitter (NBS). One beam is used to measure the pump energy by the energy meter (EM, MAESTRO, Gentec Electro-Optics Inc.). The other beam is focused on the sample by a cylindrical lens (CL) to form an excited stripe. The emission spectrum is collimated by a convex lens (*L*) and split to two sub-beams with same energy after filtered the pump light. One beam is focused by a fiber optic collimator into the fiber probe of the fiber spectrometer (FS, AvaSpec-ULS2048CL-4-EVO) and measured by the software (AvaSoft 8). This is the first part of the experimental setup: random lasing measurement. The second part of the experimental setup is speckle-free imaging using the random laser as the light source. A 1951 US Air Force (AF) resolution test chart is illuminated by the other beam of the random laser. Its details can be seen in Supporting Information (Figure S4, Table S1). A 20× objective lens is used to collect the signal to the CCD coupled spectrometer (Retiga R6, Teledyne Imaging). Two scatter films (S1 and S2) are used to simulate the scattering environment.

## 3 Results and discussion

### 3.1 Radiation characteristics of the random laser

**Figure 2:**
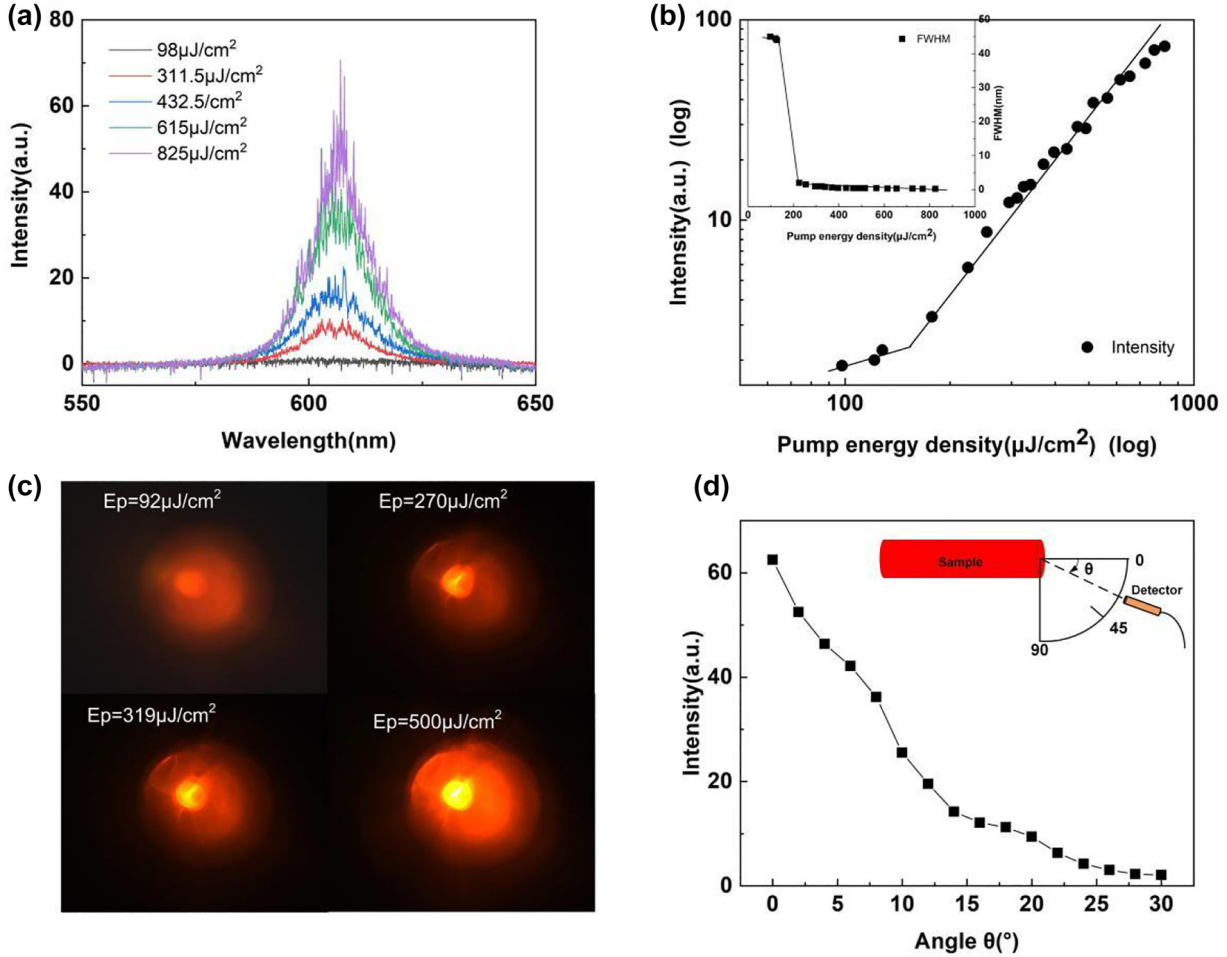
Radiation characteristics of the random laser. (a) The emission spectrum of the NPDDNLC sample as a function of the pump energy density. (b) The peak intensity and FWHM of the corresponding emission spectrum as a function of the pump energy density. (c) The photographs of the “hot” spots at different pump energies. (d) The peak intensity of the emission spectrum as a function of the angle *θ*.

The random lasing characteristics of the NPDDNLC sample are measured at room temperature. The emission spectrum of the NPDDNLC sample as a function of the pump energy density is shown in Figure 2(a). The peak intensity and the full width at half maximum (FWHM) of the emission spectrum as a function of the pump energy density are plotted in the Figure 2(b). When the pump energy is low, the emission spectrum is a broad spontaneous emission with the line width of about 45 nm. When the pump energy density exceeded 153 μJ/cm^2^, some discrete sharp peaks with the FWHM of below 1 nm suddenly emerge on the top of emission spectrum. Meanwhile, the peak intensity of emission spectrum increases rapidly. The low laser threshold (about 153 μJ/cm^2^) of the random laser can be evaluated easily by the curve knee of the peak intensity as a function of the pump energy density as shown in the Figure 2(b) and also can be evaluated by the curve knee of the FWHM as a function of the pump energy density as shown in the inset of Figure 2(b). When the pump energy is about five times of the threshold energy, the FWHM of the peaks can reduce to 0.23 nm as shown in the Figure S5(a). Since there is no predefined cavity structure, the laser behavior might results from the multiple scattering caused by the random scatter centers including TiN NPs and NLC molecules. Due to the multiple scattering, multimode is the characteristic of random lasers rather than single laser mode. Moreover, the random laser is enhanced significantly by the LSPR of TiN NPs. Figure 2(c) shows the photographs of the sample at different pump energies. When the pump energy is lower than the lasing threshold, there is no “hot” spot in the NPDDNLC sample, which indicates that no random laser occurs under this pump energy. When the pump energy density is 270 μJ/cm^2^, a small “hot” spot appears in the sample, which indicates that the random laser has been generated. With increasing the pump energy, the “hot” spot area will increase. As a result, the number of random laser modes increase as the pump energy increases (see Figures 2(a) and S5(b)). Figure 2(d) shows the peak intensity of the emission spectrum as a function of the angle *θ*. The angle *θ* is defined as the included angle between the detecting direction and the direction of the capillary tube as shown in the inset of Figure 2(d). As we can see in the Figure 2(d), the intensity of the emission spectrum is mainly confined in an angle range from 0° to 15°. The direction emission of light is very advantageous for improving the brightness of imaging. In addition, the emission direction of random laser is easy to be artificially controlled.

**Figure 3:**
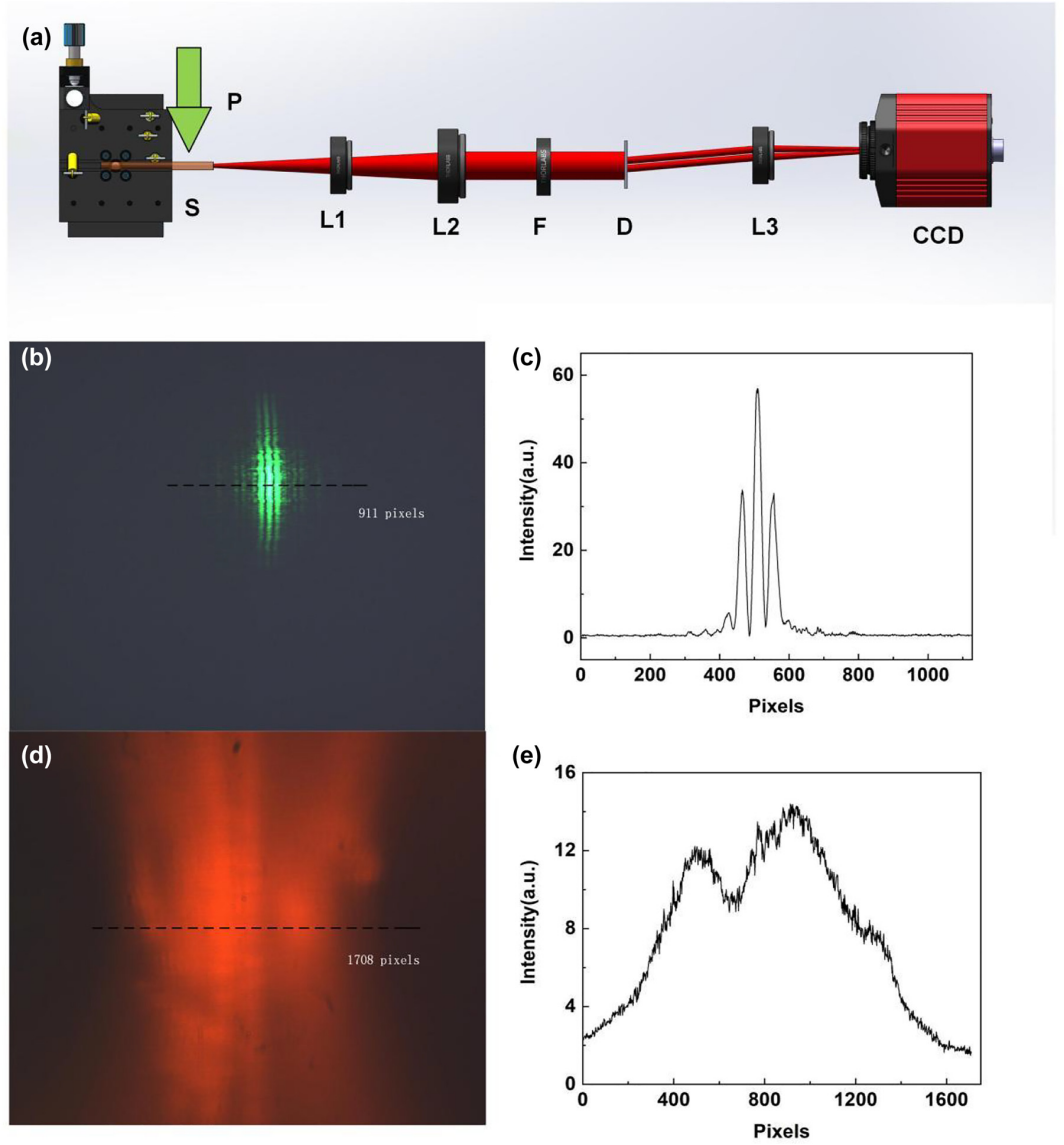
Spatial coherence. (a) Schematic diagram of the experimental setup. P, pump laser; S, NPDDNLC sample; L1, convex lens 1; L2, convex lens 2; F, filter; D, Young’s double slit plate; L3, convex lens 3. Far-field interference fringe images of (b) Nd:YAG laser and (d) NPDDNLC random laser. (c and e) The intensity of the interference fringe on the dotted path.

As we can see in Figure 2, lots of laser modes are generated by the NPDDNLC sample when the pump energy exceeds the laser threshold, and the lasing modes come from “hot” spots in different locations. As a result, the spatial coherence of the NPDDNLC random laser should be low. The spatial coherence of a conventional laser and the random laser are compared as shown in the Figure 3. The schematic diagram of the experimental setup using random laser as light source is shown in Figure 3(a). And, the schematic diagram of the experimental setup using Nd:YAG laser as light source can be seen in Figure S6(a). The far-field interference fringe image of Nd:YAG laser shows a pattern of stripes that alternates distinctly between bright and dark as shown in Figure 3(b). However, the far-field interference fringe image of random laser does not appear obvious light and dark interference fringe as shown in Figure 3(d). The spatial coherence *γ* can be expressed as the formula $\gamma =\left(I_{\max}-I_{\min}\right)/\left(I_{\max}+I_{\min}\right)$ , where, *I*_max_ is the intensity of bright stripe, *I*_min_ is the intensity of dark stripe. According to the data in Figure 3(c) and (e), the spatial coherence *γ* of the Nd:YAG laser and random laser is about 0.96 and 0.15, respectively. The low spatial coherence of random laser is very advantageous for high resolution imaging.

### 3.2 Imaging in scatterless environments

**Figure 4:**
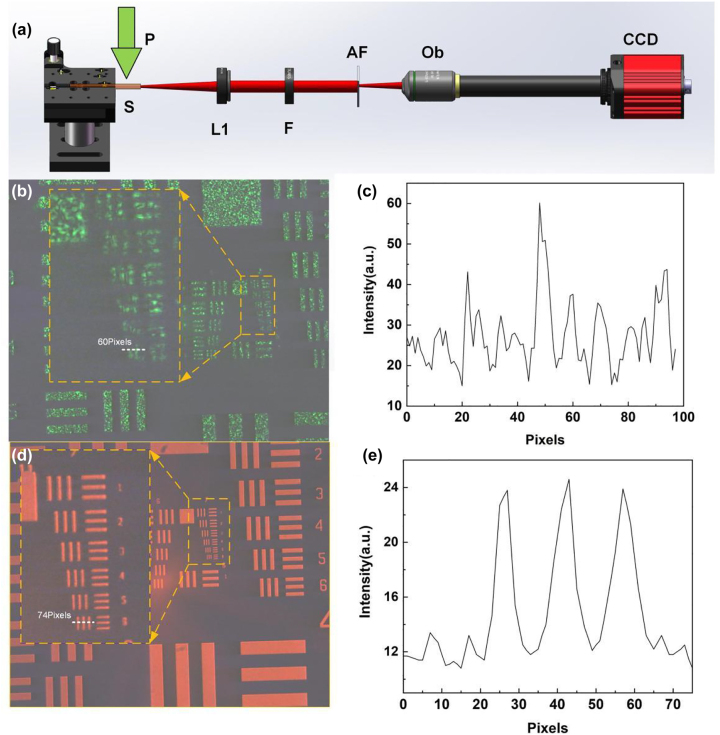
Imaging in scatterless environments. (a) Schematic diagram of the experimental setup. P, pump laser; S, NPDDNLC sample; L1, convex lens 1; F, filter; AF, AF resolution test chart; Ob, microscope objective. Images illuminated by the (b) Nd:YAG laser and (d) NPDDNLC random laser. (c and e) The intensity on the white dotted line.

Next, the ability of the NPDDNLC random laser to produce high resolution imaging is demonstrated in scatterless environments. A 1951 US Air Force (AF) resolution test chart was tested by using the NPDDNLC random laser and the Nd:YAG laser as laser source, as shown in Figure 4. The resolution of the image is defined as the limit to which adjacent lines can be distinguished and can be obtained by looking up the table shown in Table S1. Figure 4(a) shows the schematic diagram of the experimental setup using random laser as laser source. And, the schematic diagram of the experimental setup using Nd:YAG laser as laser source can be seen in Figure S6(b). Because of the high spatial coherence of the Nd:YAG laser, interference fringes will appear in the image, which seriously damages the quality of the image. When the spacing of the lines less than a certain value, the adjacent lines cannot be distinguished as shown in Figure 4(b) and (c). The results show that the resolution of the image illuminated by the Nd:YAG laser is about 32 lines/mm. Since the NPDDNLC random laser is a low coherence laser source (*γ* = 0.15), the resolution of the image illuminated by the random laser is very high as shown in Figure 4(d) and (e). Due to the limitation of our AF resolution test chart itself, the resolution illuminated by the random laser is 228.1 lines/mm, which is the highest value of the AF resolution test chart used in this experiment. Therefore, the actual resolution is expected to be much higher than this value. In addition, the signal-to-noise ratio (SNR) can be used to quantify the image sharpness. In here, it can be approximately expressed as $\text{SNR}=\left(I_{s}-I_{n}\right)/\left(I_{s}+I_{n}\right)$ , where *I*_*s*_ is the intensity of the signal, and *I*_*n*_ is the intensity of the noise. When SNR is 0, the sharpness of the image is the worst. And when SNR is 1, the sharpness of the image is the best. According to the data in Figure 4, SNR is near 0.05, when the Nd:YAG laser is the laser source. This means that the useful information and adulterated noise can be difficult to distinguish. It’s worth noting that the SNR is about 0.38, when the random laser is the laser source. This means that one can distinguish signals easily.

### 3.3 Imaging in scattering environments

**Figure 5:**
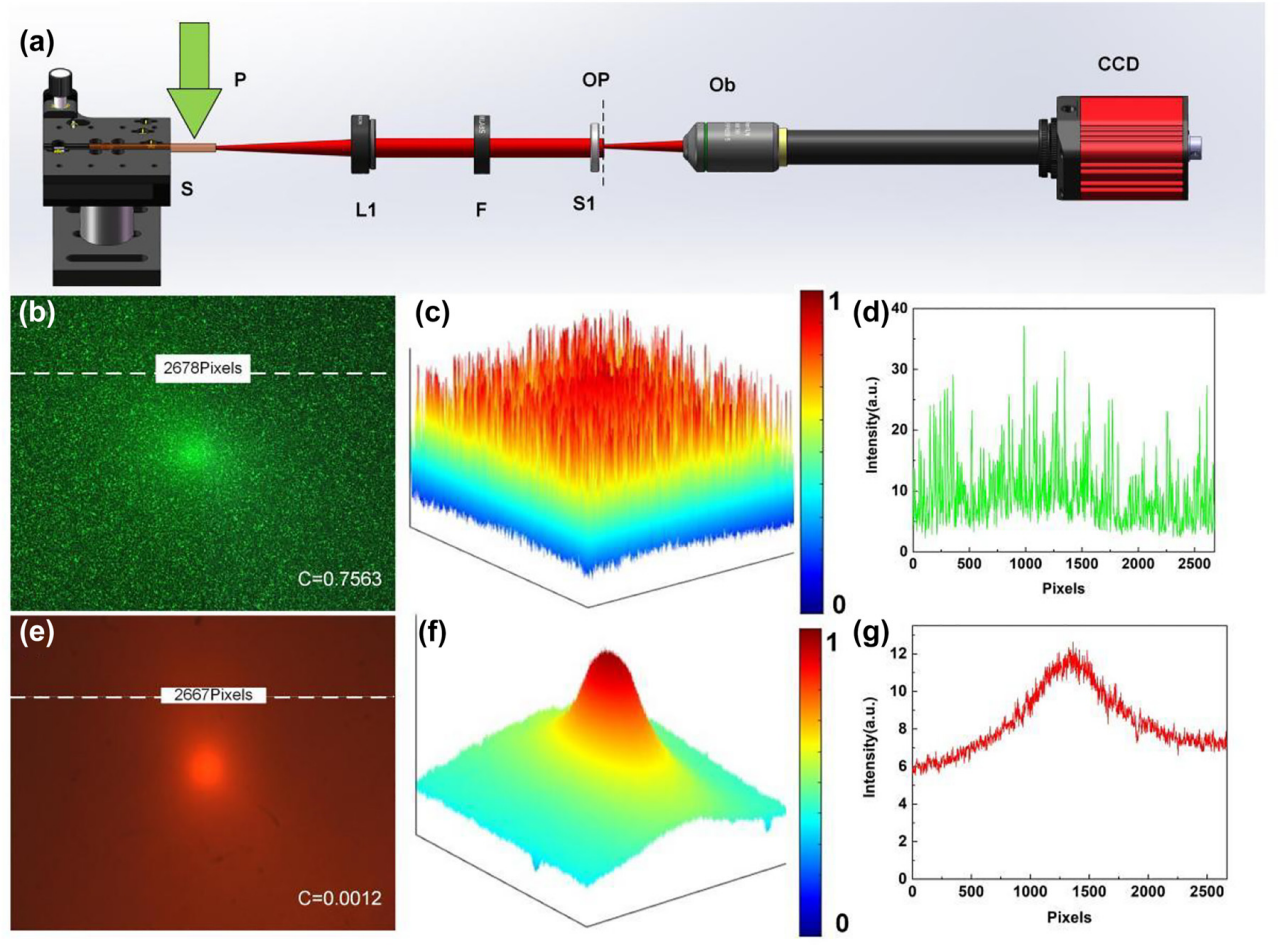
Speckle pattern in scattering environment. (a) Schematic diagram of the experimental setup. P, pump laser; S, NPDDNLC sample; L1, convex lens 1; F, filter; S1, scattering film 1; OP, object plane; Ob, microscope objective. Speckle formation illuminated by the (b) Nd:YAG laser and (e) NPDDNLC random laser. (c and f) The light intensity distribution of the corresponding picture. (d and g) The light intensity distribution along the white dotted line. The scattering film is 1500 mesh.

Then, the ability of the NPDDNLC random laser to avoid speckle formation and improve image quality is demonstrated in scattering environment. First, the NPDDNLC random laser can avoid speckle formation was demonstrated. The schematic diagram of the experimental setup using random laser as laser source is shown in Figure 5(a). And, the schematic diagram of the experimental setup using Nd:YAG laser as laser source can be seen in Figure S6(c). The strong speckle with notable intensity variation is clearly seen when using a Nd:YAG laser as shown in Figure 5(b)–(d). This is caused by the high spatial coherence of the Nd:YAG laser. When the light passes through the scatter film, the light with high spatial coherence interferes with each other to form the strong speckle. The speck contrast $C=\sigma /\left\langle I\right\rangle$ can be used to quantify the degree of speckle [17]. Where, *σ* is the standard deviation of the intensity, and $\left\langle I\right\rangle$ is the average intensity. The speck contrast *C* = 0.7563 for the Nd:YAG laser, which is detrimental to imaging applications. Fortunately, the NPDDNLC random laser can effectively avoid speckle formation as shown in Figure 5(e)–(g). The speck contrast *C* = 0.0012 for the NPDDNLC random laser is much smaller than that for the Nd:YAG laser. We further studied the speckle patterns illuminated by the random laser in different scattering environments. Results show that the speck contrast is kept very small (less than 0.02) in different scattering environments as shown in Figure S7. This is essential for high resolution imaging in the strong scattering environment.

**Figure 6:**
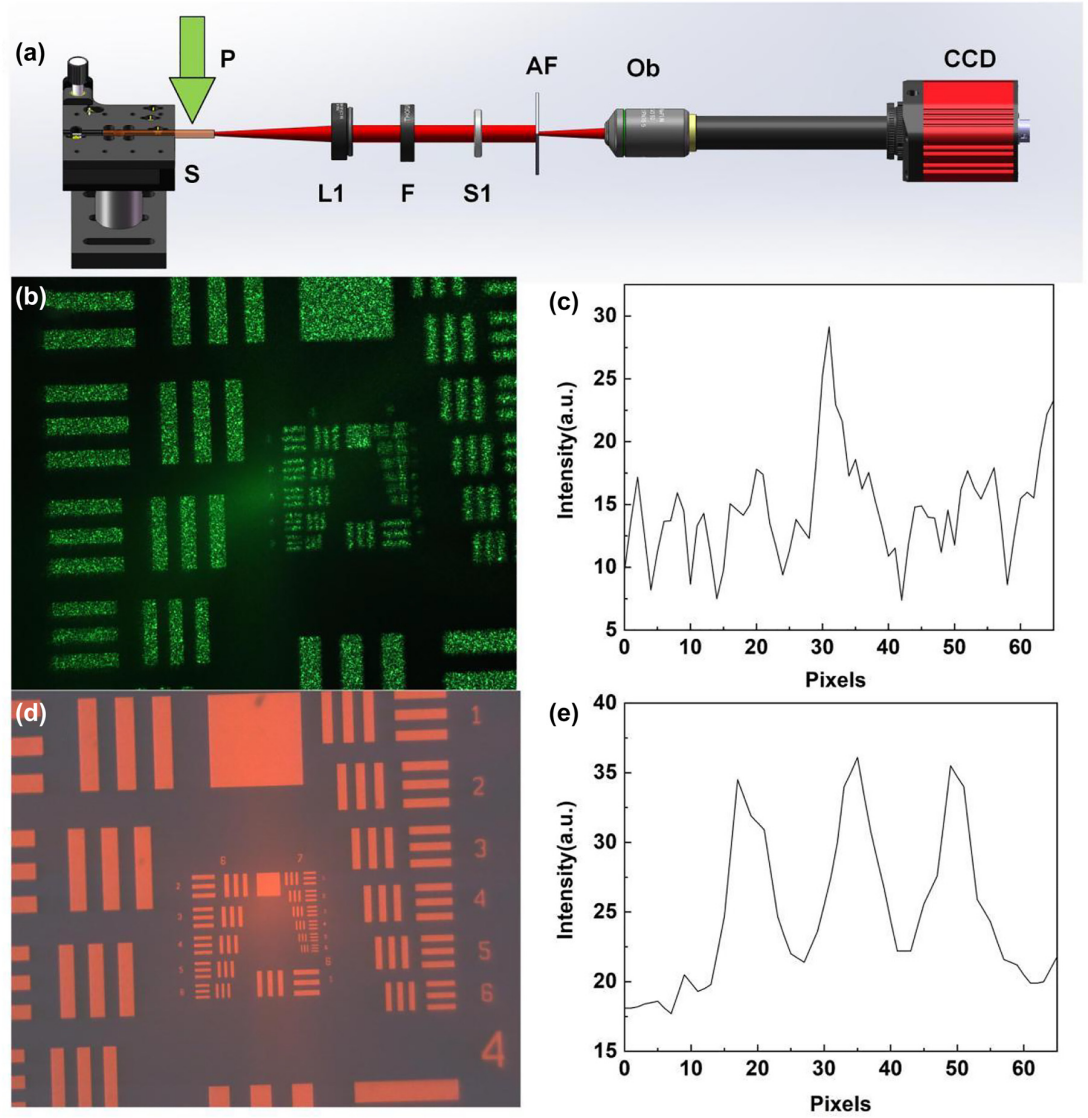
Images illuminated by the laser beam after passing through the scattering environment. (a) Schematic diagram of the experimental setup. P, pump laser; S, NPDDNLC sample; L1, convex lens 1; F, filter; S1, scattering film 1; AF, AF resolution test chart; Ob, microscope objective. AF resolution test chart images illuminated by the (b) Nd:YAG laser and (d) NPDDNLC random laser after passing through strong scattering environment. (c and e) The light intensity distribution of the bars (group number 7, element 6) in the AF resolution test chart.

Figure 6 shows the images illuminated by the laser beam after passing through strong scattering environment. Figure 6(a) shows the schematic diagram of experimental setup using random laser as laser source. The schematic diagram of the experimental setup using Nd:YAG laser as laser source can be seen in Figure S6(d). As we can see in Figure 6(b), the resolution of image illuminated by the Nd:YAG laser after passing through the scattering film is improved compared to that in Figure 4(b). This is due to the reduced spatial coherence of the laser beam after passing through the scattering film. However, strong speckle patterns are clearly visible in the bars of the AF resolution test chart. Because of the speckle, the light intensity distribution of the image is disordered, and the signal-to-noise ratio (SNR) is small, as shown in Figure 6(c), which makes it very difficult to identify the location of the bars. This severely degrades the quality of the image. Excitingly, the presence of strong scattering has little effect on the quality of the image illuminated by the NPDDNLC random laser, and no speckle was found in the bars as shown in Figure 6(d). This is mainly due to the low spatial coherence of the random laser. The light intensity distribution of the image is regular and the SNR is maintained as high as about 0.32, as shown in Figure 6(d) and (e), which allows for high quality imaging. In addition, we further investigated it in different scattering environments. Results show that the imaging quality maintains high when using the random laser as laser source in the complex scattering environments, as shown in Figure S8.

**Figure 7:**
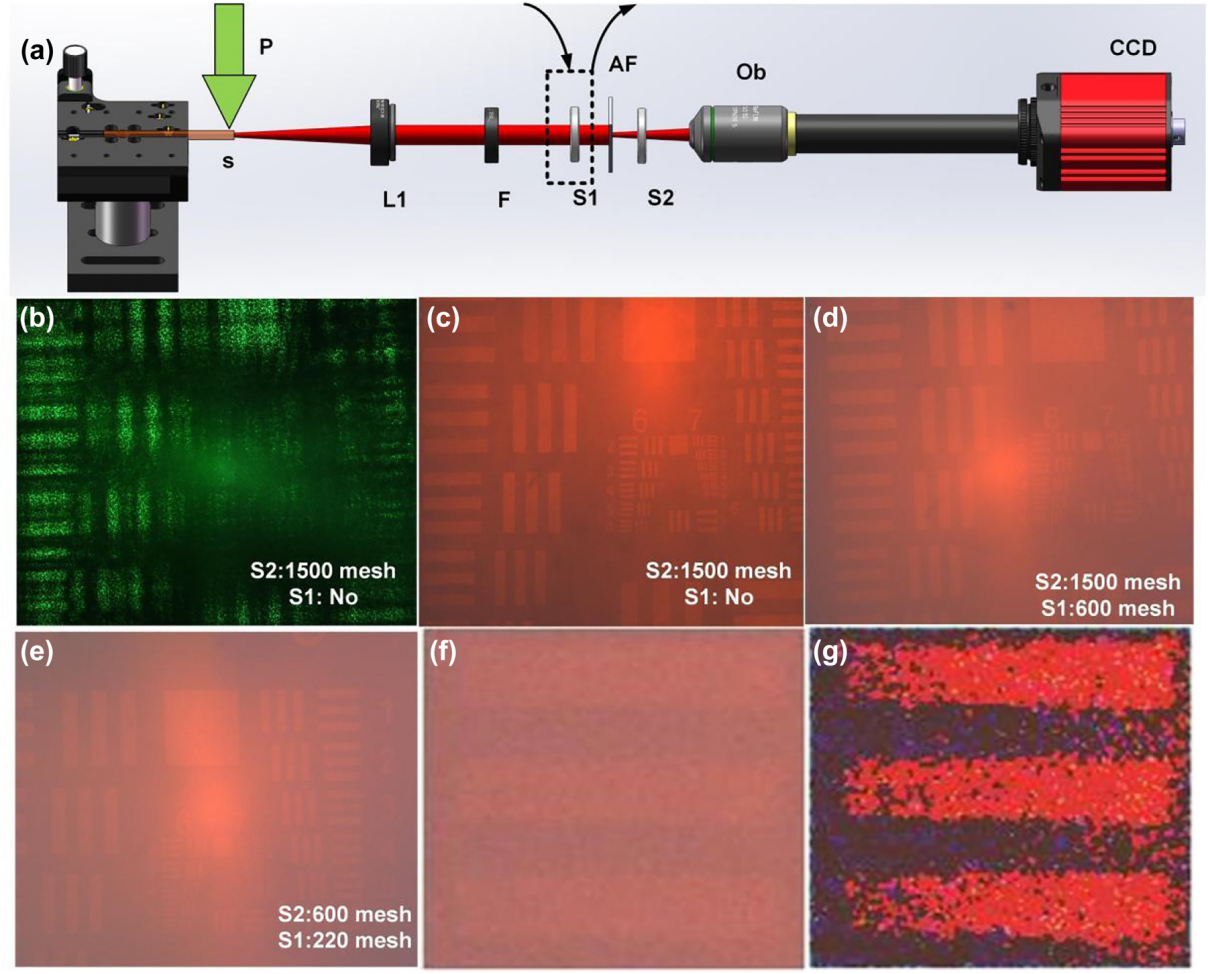
Imaging in complex scattering environments. (a) Schematic diagram of the experimental setup. P, pump laser; S, NPDDNLC sample; L1, convex lens 1; F, filter; S1, scattering film 1; AF, AF resolution test chart; S2, scattering film 2; Ob, microscope objective. Images illuminated by (b) the Nd:YAG laser and (c, d, and e) NPDDNLC random laser in complex scattering environments. (f) Original image of one element. (g) Optimized image of the element.

The robustness of image in complex scattering environments is a parameter that must be considered for imaging applications. We designed an experiment to study the robustness of images in complex scattering environments, and the schematic diagram of experimental setup using random laser as laser source can be seen in Figure 7(a). Figure 7(b) shows the image of the signal after passing through the scattering film S2, when the laser source is the Nd:YAG laser. The schematic diagram of experimental setup using Nd:YAG laser as laser source can be seen in Figure S6(e). Just as we expected, the image of the signal after passing through the scattering film (S2) will become very unclear, making it impossible to distinguish the elements of the card, as shown in Figure 7(b). However, when the NPDDNLC random laser is used as the laser source, the image of the signal after passing through the scattering film (S2) retains good sharpness and resolution as shown in Figure 7(c). In order to further demonstrate stability of the image illuminated by the random laser in complex scattering environments, we conducted imaging studies under complex scattering environments. We placed a scattering film on the front and back of the AF resolution test chart. First, the AF resolution test chart is illuminated by the random laser beam after passing through the scattering film (S1). Then, the image signal passes through the scattering film (S2). Results show that although the brightness of the image slightly varies in the different scattering environments, the image still maintains high resolution as shown in Figure 7(d) and (e). Moreover, its sharpness can be effectively improved by a simple algorithm, as shown in Figure 7(f) and (g). For simplicity, let’s take one element as shown in Figure 7(f) and process it. First, we calculate the average intensity value of the image. Then, the intensity of each pixel is compared with the average value. If the intensity value is greater than the average value, multiply it by 1.2. Otherwise multiply it by 0.5. In this way, the brightness of each pixel can be enhanced or weakened according to the relationship between the value and the average value.

## 4 Conclusions

In summary, our study successfully showcased the capabilities of a random laser enhanced by TiN nanoparticles as a unique light source that possesses narrowband, sufficient brightness, and low spatial coherence. This light source proves to be highly beneficial for speckle-free imaging applications, particularly in complex scattering environments. The low spatial coherence of the random laser beam eliminates the presence of distinct speckle effects, with a speck contrast of less than 0.02, which is imperceptible to the human eye. Moreover, we conducted a comprehensive investigation on the imaging performance when utilizing the random laser enhanced by TiN nanoparticles as the laser source in complex scattering environments. The results demonstrate that both in scatterless and complex scattering environments, the random laser outperforms traditional lasers in terms of image resolution and sharpness. This systematic analysis confirms the effectiveness of the random laser enhanced by TiN nanoparticles as an outstanding laser source for robust speckle-free imaging applications. We firmly believe that this laser source holds immense potential for diverse applications, including free-space optical communication. The advantages demonstrated in this study pave the way for future advancements and utilization of the random laser in various fields.

## Supporting Information

Optical properties of the DCM ethanol solution, SEM image, and optical properties of TiN NPs, the micrograph and resolution of the 1951 USAF resolution test chart, the FWHM of the random laser peaks, the schematic diagram of the experimental setup using Nd:YAG laser as light source, the speckle pattern illuminated by the NPDDNLC random laser at different scattering environments, AF resolution test chart images illuminated by the NPDDNLC random laser in the different scattering environments.

## Supplementary Material

Supplementary Material Details
