# Supplementary material for: Robust speckle-free imaging using random lasers enhanced by TiN nanoparticles in complex scattering environments
Source: Nanophotonics. 2023 Nov 2;12(23):4307–17. doi: 10.1515/nanoph-2023-0484 (PMC11502025; doi:10.1515/nanoph-2023-0484)
Supplement: Supplementary file 1 — Supplementary Material Details [file j_nanoph-2023-0484_suppl_001.docx]

Supporting Information for

**Robust** **speckle-free imaging using random lasers enhanced by TiN nanoparticles in complex scattering environments**

Yuan Wan^1,2^*, Zhihao Li^1^, Zexu Liu^1^, Yang Yang^1^, Hongzhen Wang^1^, Xianlong Liu^1^, and Yangjian Cai^1,2^*

^1^Shandong Provincial Engineering and Technical Center of Light Manipulations, Shandong Provincial Key Laboratory of Optics and Photonic Device, School of Physics and Electronics, Shandong Normal University, Jinan 250014, China

^2^Joint Research Center of Light Manipulation Science and Photonic Integrated Chip of East China Normal University and Shandong Normal University, East China Normal University, Shanghai 200241, China

*Corresponding author:

[wanyuansdnu@1](mailto:luogen@bit.edu.cn)63.com (Yuan Wan); *[yangjian_cai@1](mailto:yangjiancai@suda.edu.cn)63.com (Yangjian Cai)*

1. **Materials**

DCM was purchased from Exciton. TiN NPs was purchased from DK nano. NLCs was purchased from Slichem. All of these materials were used as received without further purification. The absorption spectrum and fluorescence spectrum of the DCM ethanol solution are shown in Figure S1. The absorption peak of DCM is about 470 nm, and its fluorescence peak is near 625 nm. The green line at wavelength of 532 nm indicates the pump light. Figure S2(a) shows the SEM image of TiN NPs. The particle size of TiN NPs is about 40 nm in diameter. Figure S2(b) shows the EDS of TiN NPs. Moreover, EDS mapping images of TiN NPs are shown in Figure S2(c-e). Results show that the material using in our experiment is high purity TiN NPs. The C and O elements measured in the Figure S2(b) are mainly from the C tape substrate and the attachment on the sample surface. In addition, The absorption spectrum, scattering spectrum and the electric field intensity distribution of TiN NP are calculated by using FDTD method. The absorption peak of the TiN NP is about 515 nm as shown in Figure S3(a). The absorption spectrum of the TiN NPs is sufficient wide, which has a considerable overlap with the absorption and emission spectrum of the DCM dye at a large wavelength range. In the wavelength range of the emission spectrum of the DCM dye, the scattering intensity of the TiN NPs is strong as shown in Figure S3(a). In addition, the electric field intensity around the TiN NPs is enhanced by the LSPR of the TiN NPs at this wavelength range, as shown in Figure S3(b, c). These are beneficial to enhance the laser properties of random laser.


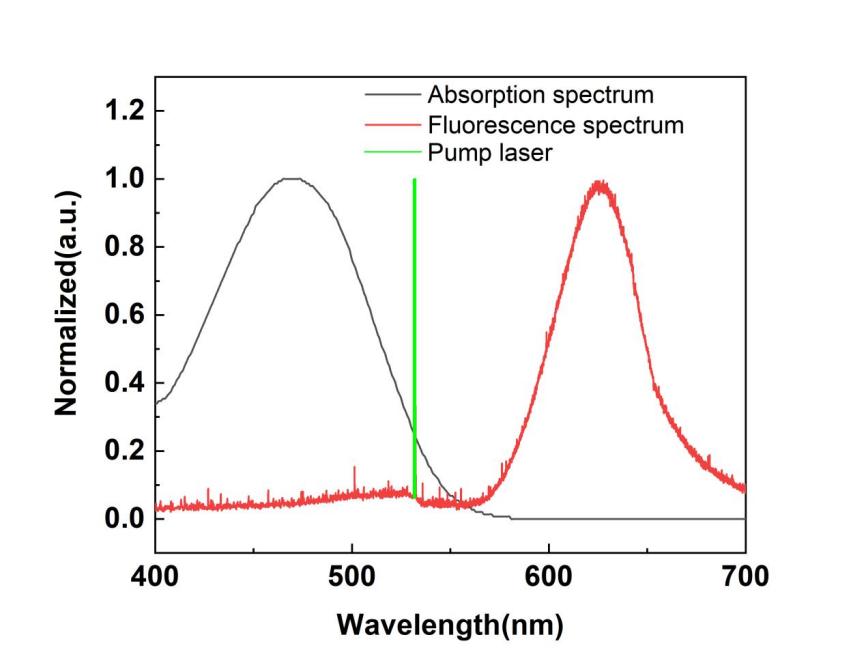


Figure S1. Optical properties of the DCM ethanol solution.


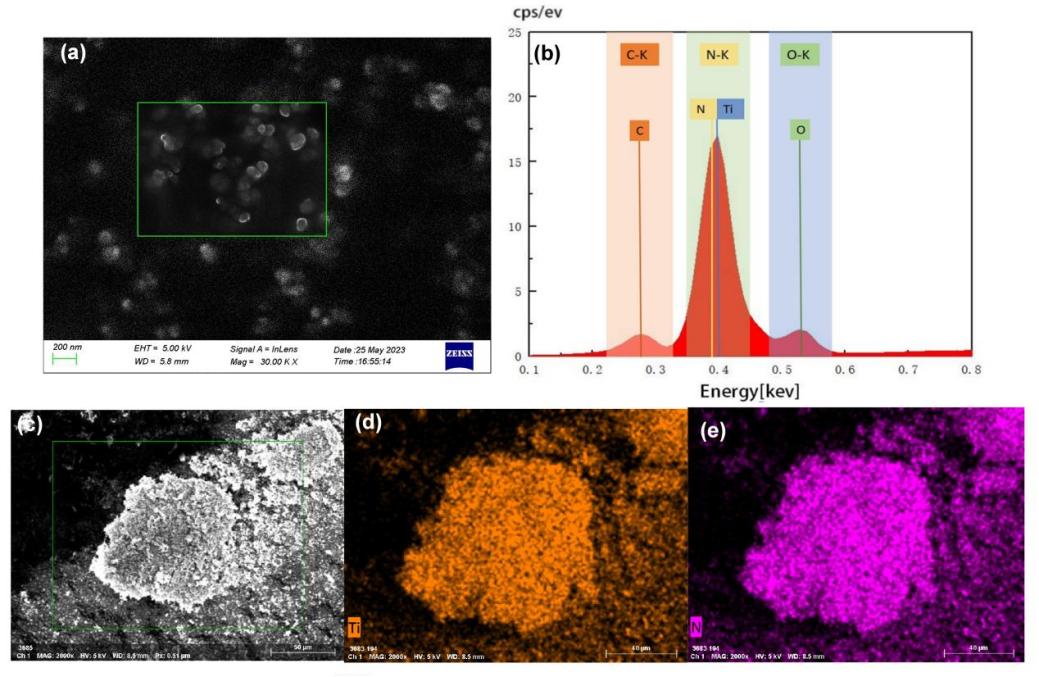


Figure S2. (a) SEM image of TiN NPs. (b) EDS of TiN NPs. (c,d,e) EDS mapping images of TiN NPs.


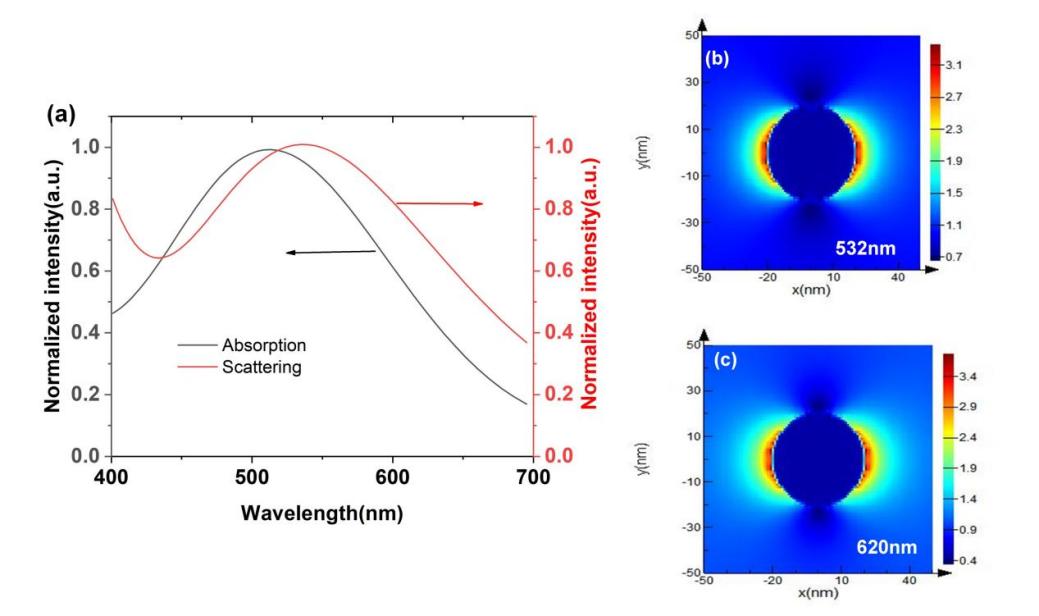


Figure S3. (a) The absorption and scattering of the TiN NPs. (b),(c) The electric field intensity distribution of TiN NP.


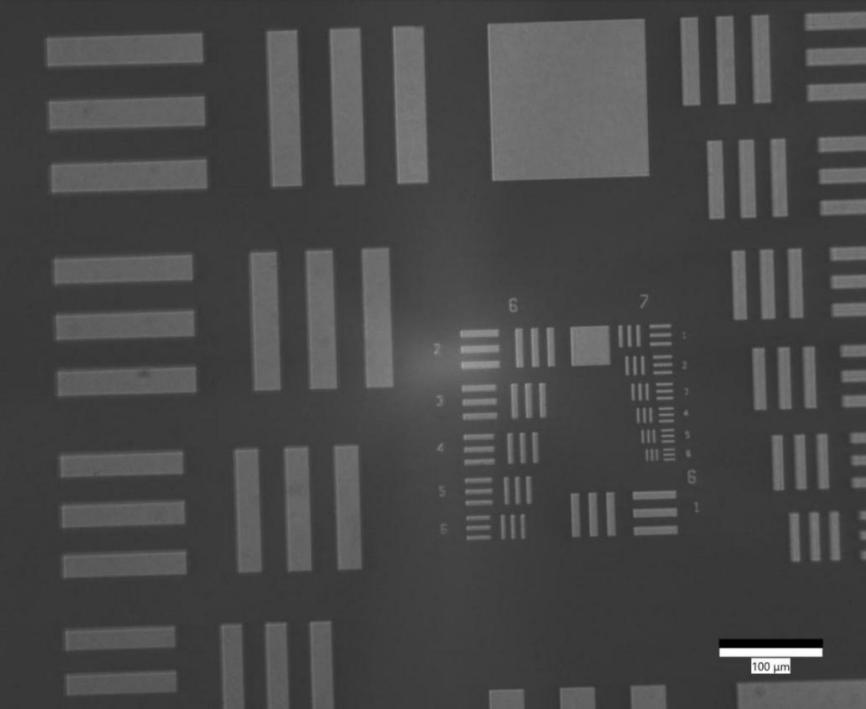


Figure S4. The micrograph of the 1951 USAF resolution test chart (Part of the test chart).

Table S1. The resolution of the1951 USAF resolution test chart.


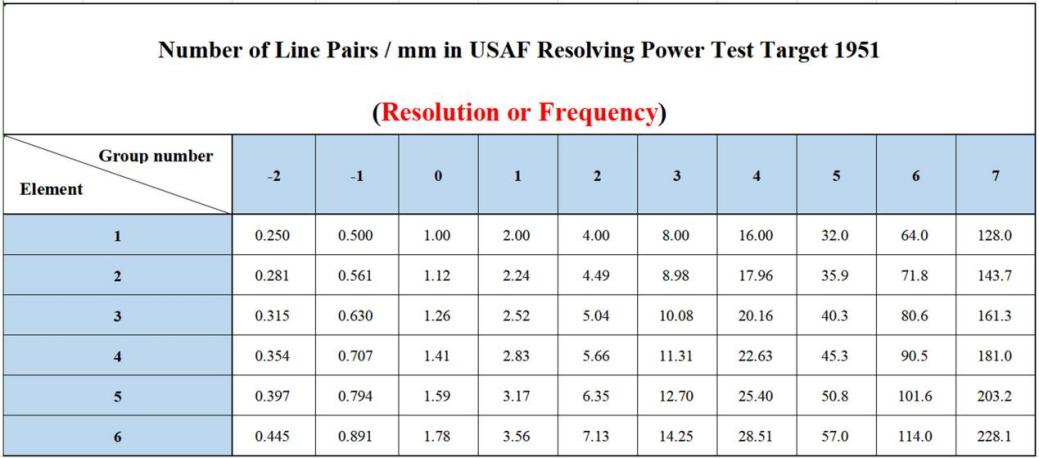


1. **Optical characteristics of random laser and its imaging applications**


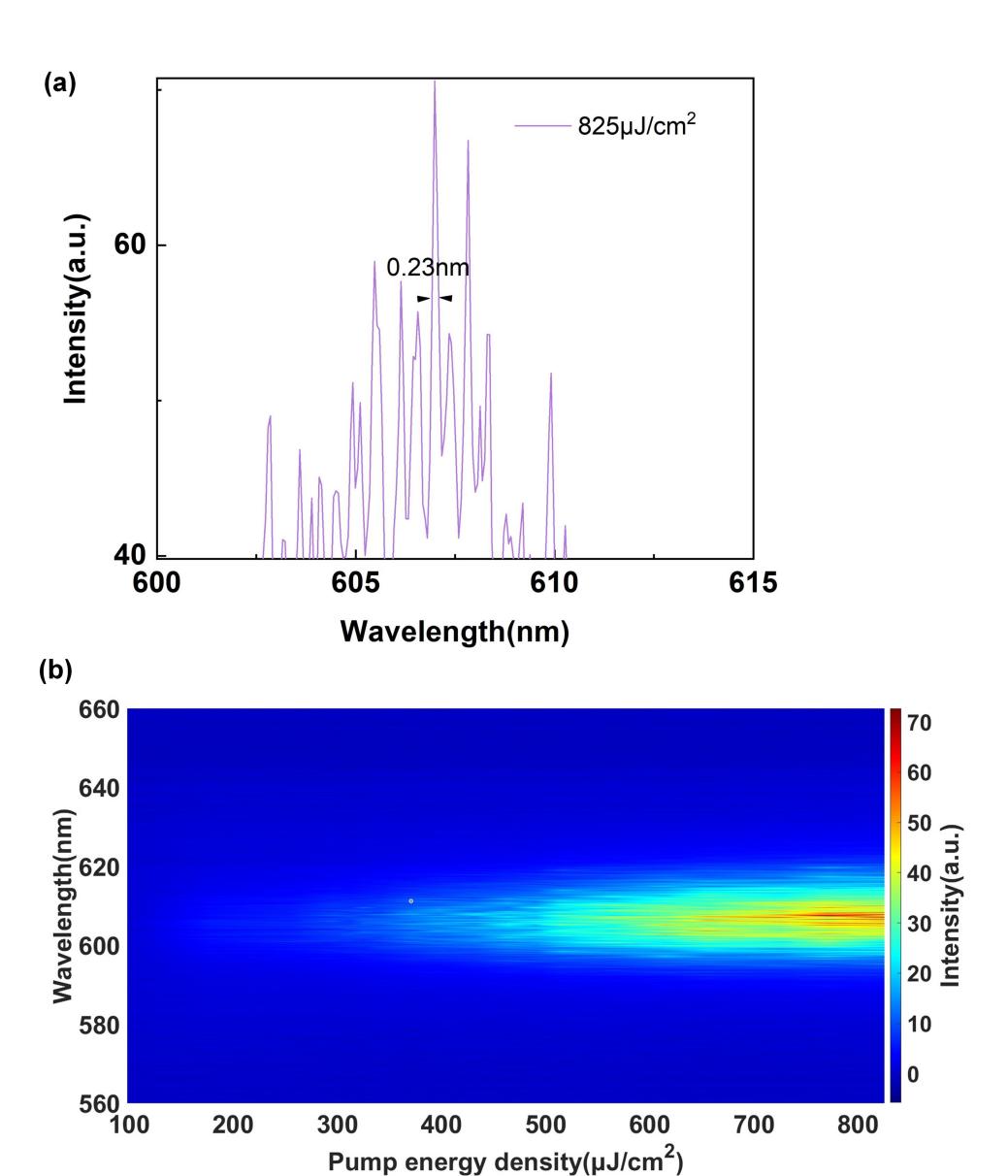


Figure S5. (a) The FWHM of the random laser peaks at the pump energy density of 825 μJ/cm^2^. (b) The intensity of the corresponding emission spectrum as a function of the pump energy density.


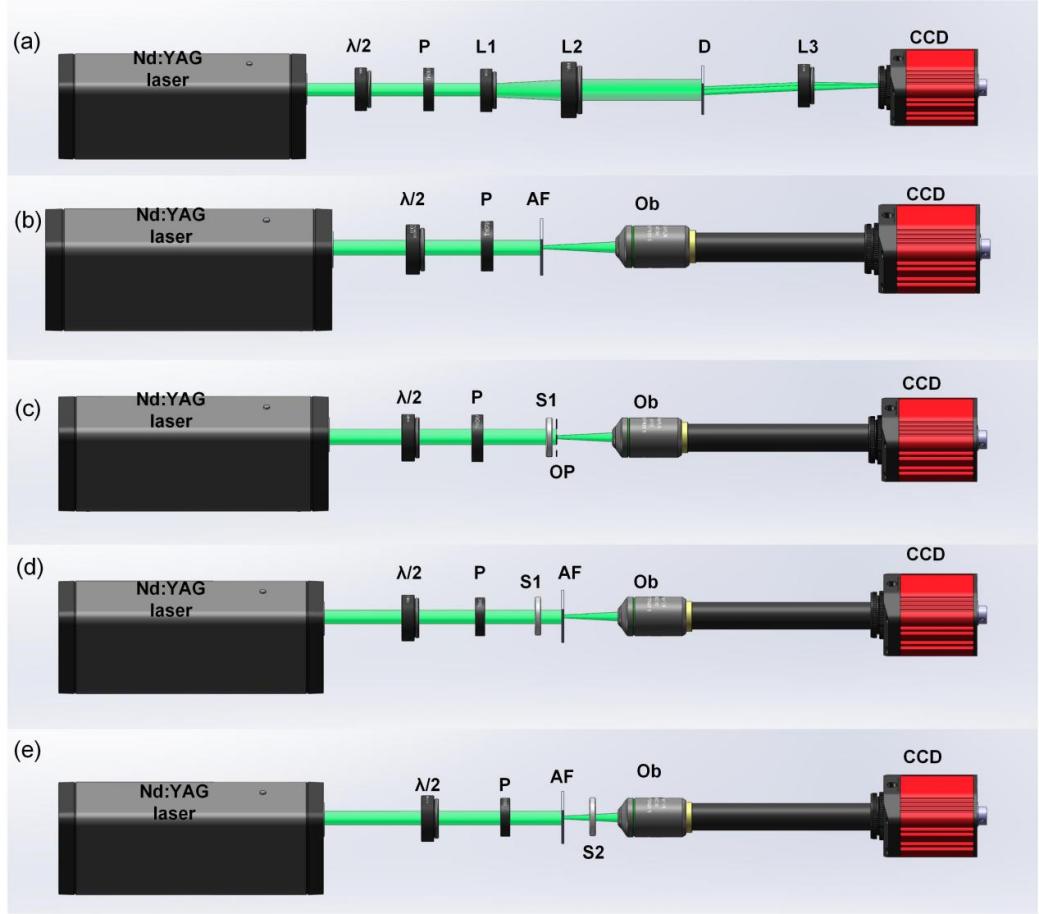


Figure S6. Schematic diagram of the experimental setup using Nd:YAG laser as light source. λ/2, half wave plate; P, polarizer; L1, convex lens 1; L2, convex lens 2; D, Young's double slit plate; L3, convex lens 3. AF, AF resolution test chart; Ob, microscope objective; S1, scattering film 1; OP, object plane; S2, scattering film 2.


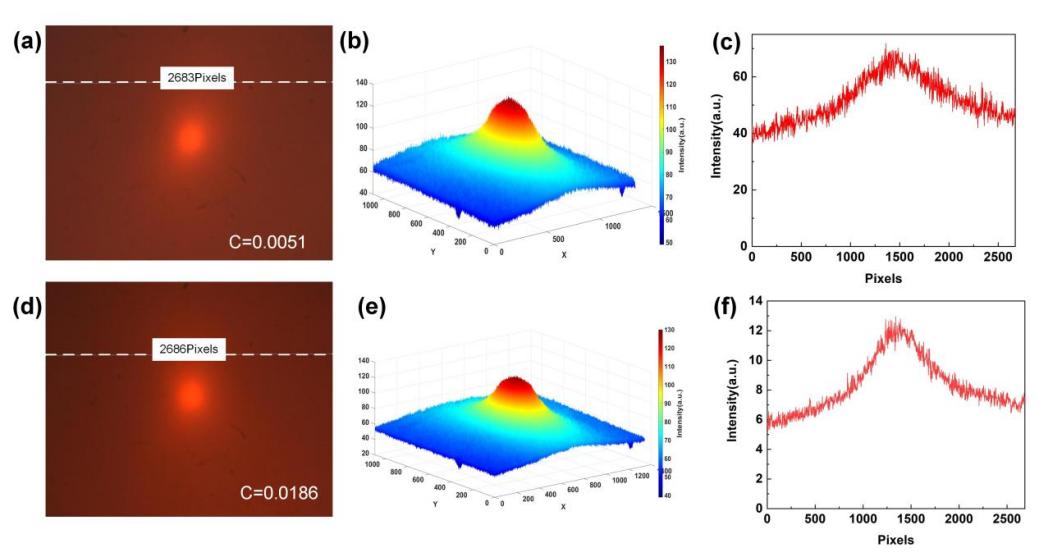


Figure S7. The Speckle pattern illuminated by the NPDDNLC random laser. (a, b, c) Scattering film is 600 mesh. (d, e, f) Scattering film is 220 mesh.


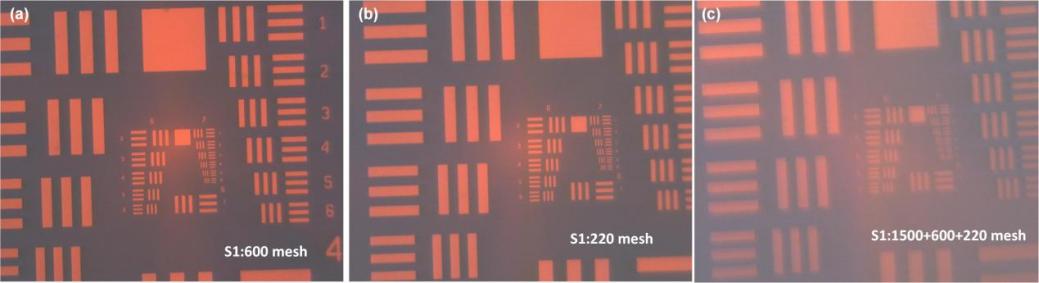


Figure S8. AF resolution test chart images illuminated by the NPDDNLC random laser in the complex scattering environment.
